# Commentary: Aesthetic Pleasure versus Aesthetic Interest: The Two Routes to Aesthetic Liking

**DOI:** 10.3389/fpsyg.2017.01197

**Published:** 2017-07-21

**Authors:** Gianluca Consoli

**Affiliations:** Ministry of Education, Universities and Research Rome, Italy

**Keywords:** pleasure, interest, aesthetic liking, processing style, elaboration

In this commentary, I draw attention to two limitations of Graf and Landwehr (2017). The article addresses one of the main questions investigated by current research on aesthetic preferences in art and product design literature: the relationship between pleasure and interest with respect to aesthetic liking (Muth and Carbon, 2016). In line with recent research (Consoli, 2016), the article stresses the multidimensional nature of aesthetic liking. In particular, according to the core premise of the Pleasure-Interest Model (Graf and Landwehr, 2015), the basic thesis is that aesthetic liking has a dual character: it can be triggered by two distinct and separate processing components, precisely a pleasure-based response and an interest-based response. In line with this thesis two studies are proposed in order to investigate if the pleasure component of aesthetic liking is triggered by a gut-level fluency experience during automatic processing, while the interest component is driven by an experience of disfluency reduction in virtue of controlled processing.

However, these studies have two relevant limitations. The first limitation is constituted by the inadequate definition and operationalization of some involved constructs, in particular “aesthetic pleasure (AP),” “aesthetic interest,” and “aesthetic liking.” The lack of clarity regarding definitions determines two main conceptual ambiguities. The first one concerns the conceptual distinction between AP and aesthetic interest. On the basis of the classical conception derived from Reber et al. (2004), AP is defined as a “pleasurable subjective experience that is directed toward an object and not mediated by intervening reasoning” (p. 2). According to Graf and Landwehr (2015), aesthetic interest also includes an affectively positive component similar to pleasure that results from the successful effort in decreasing disfluency during controlled processing. However, there is a widespread agreement that this kind of affective process represents an occurrence of AP, generally conceived as a post-insight reaction, while interest represents a pre-insight anticipation, evoked by the appraisal of high stimulation potential and the expectation of coping potential (Silvia, 2008; Muth et al., 2015; Labroo and Pocheptsova, 2016). The second ambiguity concerns the preference formation process. In Graf and Landwehr (2015), aesthetic liking is defined as the “outcome of the preference formation process.” However, there is a widespread agreement that automatic preference formation processes and controlled ones produce very different outcomes (Kahneman, 2011). Aesthetic liking is most likely more complex than conceptualized by the studies.

Accordingly, from the point of view of operationalization, it is not clear in the present study what kind of aesthetic appreciation is at stake during the different phases of the experimental procedure. Is it a form of automatic affective appraisal (non-conceptual, non-verbal, and non-systematic response spontaneously triggered by an affective reaction) or a form of controlled cognitive evaluation (deliberate judgment based on reasons)? Only the first study provides a manipulation check for the amount of cognitive elaboration. However, more rigorous tests of processing style are available. Moreover, the measures for pleasure and interest, respectively derived from Turner and Silvia (2006) and Silvia (2005), do not clarified this point. The items are verbal judgments, but they exclusively refer to subjective experience and do not asked for more extended supporting ratings. Additionally, the operationalization of gut-level fluency is questionable. In the pretest and in the first and the third phase of the procedure subjective fluency was evaluated using a three-item questionnaire with the following label: “The process of thinking about this picture is….” This seems to specifically measure conceptual fluency, not perceptual fluency—as measured in the second phase using the label “I perceive the picture to be….”

Second limitation: it is not clear which basic hypothesis the study is based on. In particular, it is possible to distinguish between two different versions, a strong version (“AP is always—or, at least, especially—triggered only by gut-level fluency and automatic processing”) and a weak version (“automatic processing is sufficient for AP—for interest, by contrast, controlled processing is required).

The strong hypothesis is not consistent with the theoretical assumptions involved in the studies. According to the quoted classical definition, AP represents a high-order phenomenal signal grounded in and function of first-order processing experience, provoked by constant self-monitoring ongoing cognitive processing, automatically elicited by internal cues associated with progress toward recognition of stimuli. So, it plays the function of meta-monitoring successful termination of both automatic and controlled processing. Moreover, this point is largely corroborated by a large body of recent evidence (Muth and Carbon, 2013; Belke et al., 2015). This evidence suggests that the extent to which perceiving challenging, and so initially unpleasant, aesthetic objects become aesthetically pleasant essentially depends on the subjects' phenomenal state of effort and cognitive mastering. AP strongly depends on the quality of elaboration in terms of extended active, and deep processing.

The weak hypothesis is compatible with comments to study 1, when authors explicitly admit: “the dashed right part [of Figure 2] from processing style to pleasure implies that participants rated the pictures as significantly more pleasant when they had processed them on a controlled processing level as opposed to an automatic processing level. This is most likely also due to the intensified interaction that occurs only during controlled processing” (p. 5). Based on the weak hypothesis, it would be interesting to also analyze the effect of disfluency reduction on pleasure which is not reported in the manuscript due to its focus on the strong hypothesis.

In sum, the theoretical framework of Graf and Landwehr (2017) and the collected evidence do not fully support the Pleasure-Interest Model and its core assumption that pleasure and interest are two independent and separate mechanisms of aesthetics response. On the contrary, it seems very plausible that, when subjects elaborate challenging and disfluent stimuli, interest, and pleasure are deeply intertwined in a self-reinforced process. From this point of view future research should specify if and how interest and pleasure, as high-order affective signals, play an intertwined anticipatory function before predictive error reduction and if and how they are integrated, after the insight, into the final liking judgment.

## Conflict of interest statement

The author declares that the research was conducted in the absence of any commercial or financial relationships that could be construed as a potential conflict of interest.
